# Will plain packaging of cigarettes achieve the expected? Perceptions among medical students

**DOI:** 10.18332/tid/154056

**Published:** 2022-10-31

**Authors:** Pinar Ay, Yesim Yasin, Osman Elbek, Murat Guner, Tanzer Gezer, Ummuhan Pece Sonmez, Murat Ceyhan, Fusun Yildiz, Elif Dagli

**Affiliations:** 1 Department of Public Health, Marmara University School of Medicine, Istanbul, Turkey; 2 Department of Public Health, Acibadem University School of Medicine, Istanbul, Turkey; 3 Clinic of Pulmonary Medicine, Kadikoy Florence Nightingale Medical Center, Istanbul, Turkey; 4 Health Institute Association, Istanbul, Turkey; 5 Faculty of Law, Kadir Has University, Istanbul, Turkey; 6 Department of Pulmonary Diseases, Dr. Suat Gunsel University of Kyrenia Hospital, Kyrenia, Cyprus

**Keywords:** plain packaging, pictorials, tobacco, qualitative study

## Abstract

**INTRODUCTION:**

Plain packaging is one of the critical strategies in eliminating the promotion of tobacco products. Evidence indicates that plain packaging decreases the attractiveness of tobacco products and enhances the effectiveness of health warnings. This study aimed to explore the perceptions of undergraduate medical students of plain packaging and new pictorial warnings before they came into use in Turkey.

**METHODS:**

This qualitative study was carried out among undergraduate students in a Medical School in Istanbul in 2019. Participants were recruited through purposive sampling, and data were collected through focus group discussions. The participants were asked to discuss their perceptions regarding one original branded pack and ten plain package models. All discussions were audiotaped and thematic content analysis was conducted.

**RESULTS:**

A total of 72 students participated in the study. None of the students had seen plain packaging before. Most of the students perceived plain packaging as more favorable compared to the branded packs. The terms used to describe plain package were: ‘appealing/desirable’, ‘attractive’, ‘beautiful’, ‘cool/eye-catching’, ‘charming’, ‘elegant’, and ‘special’. Some students indicated that they would have preferred plain packs over the branded ones if both types of products had been in the market and provided they were of the same brand. Pictorials had different impacts based on their content. At the same time, outer body deformities were perceived as ‘real’ and provoked unfavorable feelings; inner organ images were defined as ‘imaginary’ and had little to no impact.

**CONCLUSIONS:**

Plain packaging was perceived as a more attractive alternative to the conventional branded packs among most participants. We must be aware of the unforeseen effects of plain packaging among different subgroups in the new generations. We suggest using outer body deformities in the pictorials more frequently due to their higher impact.

## INTRODUCTION

Packaging is the most well-known tobacco marketing strategy in countries where advertising and promotional material are prohibited^1,2^. Framework Convention on Tobacco Control (FCTC) proposes measures to combat this strategy. FCTC Article 11 indicates that tobacco product packaging and labelling should not promote a product, and packaging should contain health warnings that explain the harmful effects of tobacco use in the form of pictograms^3^. Health warnings and pictograms should be large, clear, visible, legible and culturally appropriate. FCTC, through Article 13, also ensures that advertising, promotion and sponsorship of tobacco products should all be banned^3^.

Plain packaging is proposed as a key measure to adopt the implementation of Articles 11 and 13 of FCTC. With plain packaging, the use of logos, colors, brand images and promotional information on the packaging are prohibited. Also, product names are displayed in standard color and font styles^4,5^. So plain packaging is expected to decrease the appeal and attractiveness of packages and eliminate the effects of advertising and promotion on the packaging^5-7^. Plain packaging is also expected to increase the noticeability and effectiveness of health warnings and reduce industry package design techniques that present some products as less harmful^5-7^.

Studies evaluating the effectiveness of plain packaging on smoking prevention and cessation yield relatively consistent evidence^6-8^. Plain packaging is reported to reduce the appeal of tobacco products and to result in a negative perception of smoking^6-8^. Plain packaging has also been shown to enhance the effectiveness of health warnings by increasing the salience of pictorials on the packs. Consequently, plain packaging is suggested to reduce initiation and experimentation, resulting in a higher motivation to quit and lower purchase intentions^7-11^.

Turkey introduced plain packaging with the amendments to Law No. 4207 on Prevention and Control of Hazards of Tobacco Products in December 2018^12^. The amendment required tobacco products to be marketed in plain packages and allowed the trademark on only one side of the pack, covering a maximum 5% of the surface area. The amendment also included an increase in the size of the pictorials from 65% to 85%. Plain packaging was put into force in January 2020, and branded tobacco products were not allowed in the market after that.

This study aimed to explore the perceptions of undergraduate medical students of plain packaging and new pictorial warnings before the amendment was implemented in Turkey. Medical students were selected as the study population because smoking is prevalent among this group; almost one in five students is a smoker in Turkey^13^.

## METHODS

### Design

This is a qualitative study which was carried out in 2019. The study protocol was developed by using the Qualitative Research Review Guidelines – RATS.

### Setting and participants

The study was carried out in a Medical School in Istanbul. Undergraduate students, who had currently been smoking, had quit and never smoked, were selected through purposive sampling and invited to participate in the study.

### Procedure

**Figure 1:**
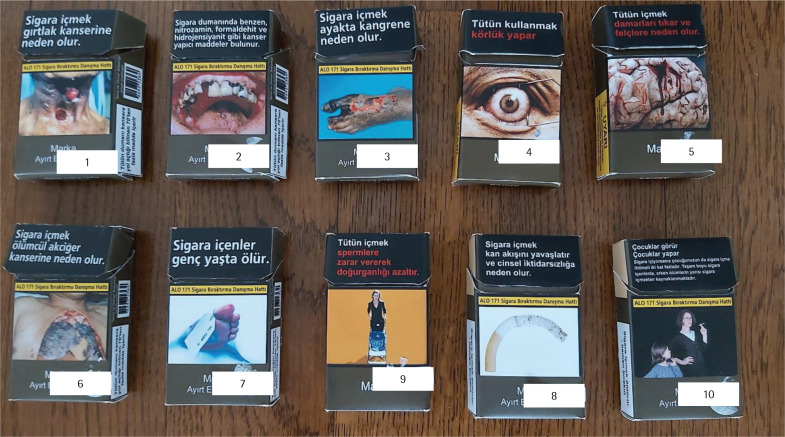
Plain package models, Turkey

Eleven cigarette packages, one original branded pack and ten plain package models were used in this study. The branded pack was obtained from the market. The researchers designed the plain package models since plain packages were not available in the Turkish market at the time of the study (Figure 1). The colors, font styles, the trademark size and pictograms were formed in line with the Regulation on the Procedures and Principles Related to the Production Methods, Labeling and Surveillance of Tobacco Products^14^. The models did not have brand names; only the word ‘brand’ was printed on the packs with the font and size specified by the amendment. The color was dark green, and the pictorials appeared on both sides of the packs as required by the new regulation. There were no cigarettes inside the packages. The plain packages released to the market soon after our study were very similar in design to the models we used in this research.

Data were collected through Focus Group Discussions (FGDs). Each focus group was formed homogenously in terms of the student’s smoking status and clinical phase (preclinical/clinical). FGDs comprised 6–8 participants and were carried out with a moderator and an observer around a round table. A semi-structured interview guide was used. FGDs were initiated with a general discussion on smoking history and motives for choosing a cigarette package. Then, each box was presented and the group members were asked to discuss their perceptions and compare the branded and plain package models. Also, the impact of each pictorial on plain packages was evaluated. Eleven FGDs were conducted until the data reached saturation.

### Data analysis

All FGDs were audiotaped after the participants provided informed consent. Recordings were transcribed verbatim and thematic content analysis was conducted. Two researchers read the transcripts several times, and identified and coded the idea elements. The codes were discussed, revised, and grouped into themes with a subgroup of authors, and the final coding framework was developed. Texts were coded with the identified themes, and an inductive approach was used. Disagreements were resolved with the subgroup of authors through consensus.

## RESULTS

A total of 72 students participated in the study; 28 were female, and 41 were final year students. Among them, 50 were current smokers, 9 were ex-smokers, and 13 were non-smokers. The age of the participants ranged 18–26 years with a mean of 22.1±2.0 years.

### Perceptions about plain packaging

The students were not familiar with the term plain package. Few students had heard the term ‘plain packaging’ before, and none had seen one. Plain packaging was perceived as more favorable compared to the branded cigarette packs by most of the students. A positive perception was expressed concerning the aesthetic look; the participants defined the design of plain packs as ‘appealing/desirable’, ‘attractive’, ‘beautiful’, ‘cool/eye-catching’, ‘charming’, ‘elegant’, and ‘special’. A student indicated that the appealing features were related to the ‘minimalist’ design of the packs. The elementary figure created a stylish look that was in line with the world’s new trends, whereas the branded packs were perceived as ‘old fashion’:

Participant: *‘I like it more (referring to the plain package), to be honest… It has a non-eye-straining, more minimalistic design; it makes me drawn/interested.’*

Moderator: *‘Minimalist design? Do you find it aesthetic?’*

Participant: *‘And the world is now … Yes. I think these things are wrong when the world is going to minimalist designs … Because minimalism is ahead of fanciness both in advertisements and in products. This kind of design (plain packaging) wouldn't be beneficial (for tobacco control).’* (Male, smoker)

The aesthetic appeal created a positive image regarding the quality of cigarettes; the products in the plain packs were evaluated as ‘good quality’ and ‘reassuring’. And the quality of the product served as an identifier of the user. Plain packages were defined to serve as a symbol for high-class or elite groups, while branded packs were accessible to everyone. The terms used to describe the aesthetics of plain packages, product quality and perceived smoker identity are listed in Table 1. Some of their comments included:

*‘This (referring to the plain package) gives an image as I smoke, but I'm not an addict, I know my limits, I have a sports car, it evokes such messages… I mean, I know how to dress and which cigarette to choose, this is the classical cigarette…’* (Male, smoker)

*‘The red one (referring to the branded package) has an image as if everyone could get/buy it, but the darker one (referring to the plain package), I don’t know how to say, looks more unique.’* (Female, ex-smoker)

Some of the students, mostly the girls, indicated that they liked the dark green color of the plain packages. A female smoker said that the dark green color reminded her of ‘olives’, and she had associated this color with being ‘healthy’.

### Purchase intentions about plain packaging

The positive perception of the plain packages was transferred to the product quality and reflected purchase intentions. If both types of products had been in the market, the students mainly indicated that they would have preferred plain packs over the branded ones, provided they were of the same brand:

Moderator: *‘What if they're both (referring to the branded package and the plain pack) on the shelf, which one would you choose?’*

Participant: *‘It actually depends on its brand.’*

Moderator: *‘Suppose they are the same brand, the same brand that you smoke, just look at the design.’*

Participant: *‘I suppose I'd take the second one [referring to the plain package].’* (Male, smoker)

### Perceptions about the pictorials

The participants indicated that the pictorials on the plain packages were more eye-catching and vivid, compared to the branded ones. The visibility made the health warnings more ‘striking’ on plain packages. Some participants indicated that the presence of pictorials on both sides of the packs and the textual warnings appearing on the lid were disturbing:

Participant: *‘I would buy the red pack [referring to the branded package] because, for some reason, pictorials on both sides [referring to the plain package] disturbed me.’*

**Table 1:** The terms used to describe the aesthetics of plain packages, product quality and perceived smoker identity (Medical students, Turkey 2019)

*Terms related to the aesthetics of the package*	*Terms related to the perceived product quality*	*Terms related to the perceived smoker identity*
Appealing/desirable	Good quality	Classy/high class
Attractive	Reassuring	Elite
Beautiful		
Cool/eye-catching		
Charming		
Elegant		
Special		

Moderator: *‘Are you disturbed because the pictorials are on both sides of the packs?’*

Participant: *‘Yes, I don't know why I felt uncomfortable … Also, the (text) warning, is on the lid, because it is on the top [on the plain package]. I could have bypassed the warning on the red pack [referring to the branded package] without reading it. The warning appearing on the lid attracted my attention [referring to the plain package] seeing it [the warning] every time I open up the pack would disturb me …’*

Moderator: *‘Do you see any other differences [between the branded and the plain packages]?’*

Participant: *‘The pictorial offends the eye more in this one [referring to the plain package].’* (Female, exsmoker)

Pictorials were observed to have diverse effects based on their content. Most of the participants indicated that pictorials of physical deformities visible on the outer body were very disturbing. Some of the students indicated that this was related to perceiving visual appearance as more important than health in the short-term. Also, students stated that they had actually seen patients with such outer deformities in the course of their lives. So, they had ‘related’ these pictorials to exposures to similar patients and labelled them as ‘real’. The pictorials with a tracheotomy opening, damaged teeth and foot gangrene (Figure 1, pictorials 1–3) were listed under this category. On the other hand, pictorials presenting inner organ pathologies were perceived as more ‘intangible’:

*‘I think people give much more importance to their appearance than their health, every day we brush our teeth, comb our hair, put on makeup, we are careful about our image …The impact (of smoking) on the image is effective in the firsthand and in the short run.’* (Female, smoker)

*‘This picture [**Figure 1**, pictorial 1] is very compatible with life, reality. Today [in our daily life] we can see such people. For example, I've seen someone like this when I was young, it still lies in my subconscious. This always triggers me in a negative way against smoking. So, I think that picture has an above average [disturbing] effect.’* (Male, smoker)

The pictorial about blindness (Figure 1, pictorial 4) was the only outer body image that did not bring a disease to mind. Most students did not associate blindness with smoking because they were not fully aware that smoking could damage the eyes. Others indicated that blindness could develop only after a very long duration of smoking. Still, the pictorial was mainly evaluated as effective because it gave the impression of being observed/watched while carrying on an unacceptable behavior such as smoking:

*‘Blindness can develop in the very long-term. Those who see this image [Figure 1, pictorial 4] can say to themselves that there are a lot of people smoking, but who gets blind? I don't think [this pictorial] is effective…’* (Female, smoker)

*‘…the image [Figure 1, pictorial 4] gives the feeling of being watched, it awakens a change in the self… it makes you feel that you are doing something bad.’* (Male, smoker)

Pictorials showing inner organ pathologies such as brain hemorrhage (Figure 1, pictorial 5) and various lung deformities were perceived as less disturbing. The participants indicated that these images were not recognized as actual parts of the body or an organ system. The students believed these pictorials were ‘fictitious’ and did not reflect ‘real-life’ situations:

*It [Figure 1, pictorial 5] appears like a poor-quality horror film image to me, it is made with Photoshop, and I laughed at it; it didn't seem scary; it seemed funny.’* (Female, smoker)

However, if the internal organ was pictured with its connection to the outer body surface, then it was also perceived as disturbing. The image, which was referred to as the ‘autopsy lung’ by the students (Figure 1, pictorial 6), had a strong impact:

*‘…you know, for example, the one with the lung [Figure 1, pictorial 6], I think it was the previous one; the autopsy lung was more impressive.’* (Male, smoker)

The pictorials that presented the impact of smoking without displaying the image of the affected organ (Figure 1, pictorials 7–9) were defined as ‘illusionary’. Also, pictorials that didn’t reflect culturally familiar people from the Turkish community (Figure 1, pictorial 10) seemed fictional. The students stated that they did not feel ‘connected’ to such images:

*‘Such pictures look like artificial pictures. Maybe they are real, but they look like cover art, film poster, so artificial ... This one [Figure 1, pictorial 7] seems to have like light effects, it has a cinematographic image…’* (Male, smoker)

*‘There's no one who looks like me [in Figure 1, pictorial 10]. Or, for example, as my friends say, it's not something I'm connected to, based on my personal experience…’* (Female, smoker)

## DISCUSSION

This study shows that plain packaging was perceived as a more attractive alternative to conventional branded packs, among most of the participants. Some of the students stated that they would have preferred to purchase cigarettes in plain packs rather than branded ones, provided that they were of the same brand. The features attributed to the plain packs mainly were linked to the perceived good aesthetics. The students indicated that these packs had a ‘minimalist’ design, an ‘elegant’ look which was in line with the recent trends. Package design was also perceived to indicate the quality of the tobacco product and the user. The cigarettes in plain packages were perceived as being of good quality and smoked by ‘elite’ groups.

There is considerable evidence that plain packaging has less appeal and a poorer image than the branded packs, in adolescents and adults among diverse populations^7-10,15-33^. Plain packs were perceived as being of low quality and associated with unfavorable personal attributes such as being ‘older’ and ‘less fashionable’^7^. Particularly, younger age groups had shown less appeal compared to the older ages. Our findings differ from the studies in the literature and indicate an increased appeal for plain packaging. This finding might be related to the study group; to our knowledge, this is the first qualitative study reporting the perception of plain packs in medical students. Plain packaged products might be the medical students’ way of differentiating themselves from the ‘ordinary’ and ‘old-fashioned’ smokers in the community. The quote about plain packaging giving ‘an image as I smoke, but I'm not an addict; I know my limits’ suggests the perception of being an ‘exceptional’ smoker, unlike the rest of the population. The favorable perception regarding plain packs might also be related to the shifts in cultural norms and values among young adults. Understanding the root causes of the positive feelings about plain packaging needs a deeper psychosocial approach and is certainly beyond this study. Still, these findings highlight the need to be aware of and to study the unforeseen effects of plain packaging perceptions among different subgroups in the new generations.

The color of cigarette packaging can have an impact on perceptions regarding harm and strength, thus influencing product choice^29^. Hoek et al.^34^ discuss that the color brown could lead to ‘natural’ connotations because it is used in recycled paper or the color white might remind people of some branded products which had been marketed as ‘light’. Lacave-García et al.^15^ also determined that the grey and brown pack colors were associated with more negative feelings than white. A French study indicated that gray-colored packages as the most effective options compared to brown or white packs^35^. In our study, some participants, particularly the girls, indicated that they liked the dark green color of the plain packages. A female smoker’s connotation of ‘olives’ evoked a perception of ‘healthiness’ and ‘wellbeing’. The plain colors used in the background of packaging should be tested before implementation because they can provoke unintended positive feelings depending on cultural differences^15,34,35^.

Studies mainly indicate that plain packaging increases the salience of health warnings and pictorials. The pictorials on plain packages are noticed more easily, recalled better and have a stronger impact^7,10,15,17,19,20,28-30,36,37^. Our results also indicate that the effects of the pictorials are more profound in the plain packs compared to the branded ones. Nevertheless, on the plain packages, the size of the pictorials was increased from 65% to 85%, and the pictorials were placed on both sides of the packs as stipulated by the new amendment^14^. These changes might also have contributed to the improved salience of the pictorials on the plain packages.

Our findings show that the pictorials have varying effects based on their content. Outer body deformities, which could be observed with a naked eye, evoked highly unfavorable feelings for most of the participants. Students described such pictorials as ‘real’ since they had seen and known patients with such disabilities in their daily lives. In contrast, the inner organ images were defined as ‘imaginary’, and ‘script from posters and movies’ with little to no impact. Similarly, pictorials culturally unfamiliar did not have an effect. These findings suggest that the impact is large when the students associate the pictorials with their past observations. But when the image is not recognized experientially, as in the example of the brain hemorrhage pictorial, it has little to no impact. A qualitative study conducted among socioeconomically disadvantaged smokers in Australia indicated that some messages were not part of the smokers’ experiences and were perceived as exaggerated and not ‘realistic’. The authors noted that the participants were suspicious of the harms described in the messages^11^. Another qualitative study also indicated the skepticism related to the health warnings; for some participants, the messages would serve as a warning only when they experienced it for themselves^38^. Hence, we suggest using outer body images and pictures of the internal organs with their connections to the outer body surface more frequently, to expose the reality of smoking harms.

### Limitations

Our study aimed to explore the subjective meanings attached to plain packaging among medical students, so we used a qualitative approach and recruited the participants through a non-probability sampling method. This sampling strategy prevents the generalizability of our results to a broader population. We explored only perceptions and attitudes regarding plain packaging and do not exactly know if these perceptions will be transferred to actual purchase intentions and smoking behavior. We should also note that in the FGDs, we used only one cigarette package as an example of branded packs which is quite limited given the large variability of the branded designs on the market. Still, we suggest that our results would be beneficial since they shed light on the unforeseen perceptions that might also exist in other communities. Furthermore, our results might be used for theory building in explaining plain packaging perceptions.

## CONCLUSIONS

Plain packaging is a critical public health strategy in preventing the cigarette pack from being used as a promotional and advertising vehicle. Yet this study showed that plain packaging could be perceived as a more attractive alternative to conventional packs among medical students. We should consider that plain packaging might have unforeseen and changing effects among young adults in different cultures. While pictorials on plain packages are more visible and noticeable, we suggest using outer body images and images of internal organs with their connections to the outer body surface more frequently due to their stronger impact.

## Data Availability

The data supporting this research are available from the authors on reasonable request.
